# Chemical Intolerance among Hairdressers in Denmark

**DOI:** 10.1371/journal.pone.0071241

**Published:** 2013-08-12

**Authors:** Marie Thi Dao Tran, Jesper Elberling, Sine Skovbjerg, Nikolaj Drimer Berg, Heidi Søsted, Jeanne Duus Johansen, Susan Hovmand Lysdal

**Affiliations:** 1 The Danish Research Centre for Chemical Sensitivities, Department of Dermato-Allergology, Copenhagen University Hospital Gentofte, Gentofte, Denmark; 2 Research Centre for Prevention and Health, Glostrup University Hospital, Glostrup, Denmark; 3 Research Centre for Hairdressers and Beauticians, Department of Dermato-Allergology, Copenhagen University Hospital Gentofte, Gentofte, Denmark; 4 National Allergy Research Centre, Department of Dermato-Allergology, Copenhagen University Hospital Gentofte, Gentofte, Denmark; The Ohio State University, United States of America

## Abstract

**Objectives:**

To investigate the prevalence and the severity of fragrance-related symptoms among hairdressers in Denmark compared with the Danish general population. Further, to characterize former hairdressers who are severely chemically intolerant to fragranced products in relation to sex, age and health- and work-related reasons for leaving the hairdressing profession.

**Methods:**

The study population consisted of all hairdressers who graduated from the public vocational schools in Denmark during 1985 and 2007 (n = 7840) and a random sample of individuals from the Danish general population (n = 6000). Both populations received a postal questionnaire on symptoms from inhalation of fragranced products and the resultant behavioural consequences. All former hairdressers also answered additional questions on health- and work-related reasons for leaving the profession.

**Results:**

No differences were found in the prevalence (OR = 1.0, CI = 0.89–1.14) or the severity (OR = 1.1, CI = 0.80–1.51) of symptoms from inhalation of fragranced products in hairdressers compared with the general population. Among hairdressers, however, experience of fragrance-related symptoms (OR = 1.2, CI = 1.01–1.31) and adjustments of social (OR = 1.8, CI = 1.12–2.80) and occupational conditions (OR = 2.8, CI = 1.84–4.25) were reported significantly more often by former hairdressers than current hairdressers.

**Conclusions:**

The prevalence and the severity of fragrance-related symptoms were similar in hairdressers and the general population. Former hairdressers were more affected by fragranced products than current hairdressers were. Although fragrance-related symptoms did not seem to be more frequent among hairdressers, the hairdressing profession might pose a problem for those who are chemically intolerant.

## Introduction

Chemical intolerance is commonly reported in the general population [1]–[3] and is characterized by reports of non-specific symptoms triggered by exposure to common airborne chemicals. Reports of chemical intolerance reactions are the main characteristic of the condition referred to as multiple chemical sensitivity (MCS) [4], [5]. These reactions are also frequently reported in patients with asthma and eczema [6]. The main coping strategy in individuals with MCS is avoidance of the chemicals that trigger symptoms [7], [8] and in severe cases this may lead to loss of occupation and social isolation [1], [8]. The development of MCS has been suggested to be a two-step process: 1) an initiation phase resulting from a single high-level or chronic low-level chemical exposure and 2) a triggering phase characterized by symptoms to low-level exposure [9]. However, the condition does not seem to fit within the principles of toxicology, immunology and/or allergy [10], [11], so the necessity of a precipitating chemical exposure has been questioned [12], [13]. Studies have shown that 20–58% of MCS patients are unable to identify an initiating event [13], [14]. In support of this, data from occupational clinics and environmental health centres show that the majority of referred MCS patients are less commonly employed within occupations that are presumed to involve a higher risk of frequent or intense chemical exposure such as the construction and manufacturing industry [15]–[18]. Nevertheless, selection bias in these reports cannot be completely disregarded as a possible source of error. To our knowledge, no epidemiological studies of the prevalence of chemical intolerance or MCS within high-risk occupations have yet been performed. Hairdressing can be regarded as a high-risk occupation involving daily exposure to multiple airborne chemicals from hair dyes, permanent waving solutions, hair bleaching products and fragranced products, which are reported to be one of the most frequent triggers of MCS symptoms [1], [17], [19]–[22]. Additionally, chemical intolerance is more frequently reported by women [1], [3], and this also makes the hairdressing profession a relevant occupation for studying the condition. Thus the aims of this study were 1) to investigate the prevalence and the severity of fragrance-related symptoms in hairdressers in comparison with the general population and 2) to characterize former hairdressers who are severely chemically intolerant to fragranced products in relation to sex, age and health- and work-related reasons for leaving the hairdressing profession.

## Materials and Methods

### Ethics Statement

The study was approved by the Danish Data Protection Agency. According to Danish legislation questionnaire studies do not need approval by an ethics committee.

### Study Population

The study population consisted of all hairdressers who graduated from the public vocational schools in Denmark during 1985 and 2007 and whose postal addresses were available from the Danish Civil Registration System (n = 7840). The hairdressing population received a postal questionnaire in May 2009. After two reminders, a response rate of 68% (n = 5324) was obtained. Only respondents who reported their current affiliation to the hairdressing profession were included in this report, providing a sample of trained hairdressers aged 22–69 years (n = 5239).

The hairdressers were compared with a random sample of individuals from the Danish general population (n = 6000). The general population sample was drawn from the Danish Civil Registration System among individuals aged 18–69 years and living in one of the 11 municipalities of Copenhagen. The general population sample received a postal questionnaire in March and April 2006. After one reminder, a response rate of 71% (n = 4242) was obtained. Further details on the hairdressing [23], 24 and general population [1] have previously been published.

### Questionnaires

For the hairdressing population, the questionnaire was structured with an initial question on whether symptoms were triggered by inhalation of fragranced products followed by questions on localisation or character of symptoms: 1) ‘upper airways’ (including symptoms from the eyes, nose, mouth or throat), 2) ‘lower airways’ (including pulmonary symptoms, 3) ‘headache’ or 4) ‘other CNS symptoms’ (including exhaustion and/or grogginess). Additionally, questions were included describing possible behavioural consequences of the symptoms. Lastly, all former hairdressers were asked to state whether different health complaints (hand eczema, asthma, allergy, musculoskeletal pain or other diseases), pregnancy or their working conditions had caused them to leave the profession (multiple answers possible).

Details of the questionnaire for the hairdressing [23], [24] and general population [1] have been published previously.

### Definition of Variables

Respondents from the hairdressing population with a positive answer to the question: ‘Have you ever had symptoms elicited by inhalation of perfume or fragranced products?’ were regarded as being chemically intolerant. The original questionnaire data from the general population were reanalysed to select a group of chemically intolerant individuals suited for comparison with the hairdressers. Respondents from the general population with a positive answer to the question: ‘Have you ever had symptoms elicited by perfume, aftershave or deodorant?’ were similarly regarded as being chemically intolerant. In both populations, individuals who stated that it was only the smell they disliked and that the smell did not provoke symptoms were not included in the chemically intolerant group.

Chemically intolerant individuals who reported fragrance-related symptoms from at least one of four classes of symptoms: 1) ‘upper airways’ 2) ‘lower airways 3) ‘headache’ or 4) ‘other CNS symptoms’ were divided into severity groups according to whether they had made behavioural adjustments. The category ‘mildly chemically intolerant’ was assigned if chemically intolerant individuals reported no behavioural adjustments. The category ‘severely chemically intolerant’ was assigned if chemically intolerant individuals reported at least one adjustment of social and/or occupational behaviour. Social behaviour was defined as avoidance of 1) use of public transportation, 2) social functions in the private sphere or 3) gatherings in the public sphere. Occupational behaviour was defined as 1) sick leave from work or school/college, 2) permanent leave from employment or school/college or 3) inability to work.

### Statistics

Statistical analyses were performed using PASW™ Statistics 18 software (SPSS Inc, Chicago, IL, USA) for Windows™. The comparison of sex and age between groups was done by either the χ^2^ two-tailed test or the Fisher’s exact test. The remaining analyses between groups were carried out using logistic regression analyses adjusting for sex and age. Adjusted odds ratios (ORs) with 95% confidence intervals (CIs) are presented to describe the associations. Significance levels were set at p<0.05.

## Results

Characteristics of the hairdressers and the general population are shown in Table 1. The distributions of sex and age were significantly different between the groups (p<0.001). The hairdressing population was mainly composed of women younger than 50 years of age, whereas individuals from the general population had a more even sex and age distribution as expected.

**Table 1:** Characteristics of the hairdressers and the general population.

	All hairdressers(n = 5239)		General population(n = 4242)	
	%	n	%	n
Sex				
Female	95.7	(5015)	54.2	(2300)
Male	4.3	(224)	45.8	(1942)
Age				
<30	12.5	(656)	11.8	(499)
30–39	49.4	(2590)	17.6	(748)
40–49	37.0	(1936)	24.1	(1021)
50–59	0.9	(47)	23.7	(1004)
≥60	0.2	(10)	22.9	(970)
Hairdresser status				
Current	55.7	(2918)	†	†
Former	44.3	(2321)	†	†

† Category not applicable.

Overall, 21.5% of the hairdressers and 15.2% of the general population reported experiencing fragrance-related symptoms, but no significant difference in frequency was found between the groups when adjusting for sex and age (Table 2). The majority of the hairdressers who reported fragrance-related symptoms, 634 of 643 (98.8%), also reported symptoms from at least one of the four classes of symptoms. This proportion was similar in the general population, 1112 of 1125 (98.6%) (Table 2). The report of fragrance-related symptoms was significantly more frequent among former hairdressers (22.7%) than among current hairdressers (20.5%) when adjusting for sex and age (OR = 1.2, CI = 1.01–1.31, Table 3). After adjusting for sex and age ‘other CNS symptoms’ were reported to a significantly higher extent by the hairdressers compared with the general population (OR = 1.5, CI = 1.15–1.90, Table 2). Among hairdressers, symptoms from the ‘lower airways’ and ‘headache’ were reported significantly more frequently by former hairdressers compared with current hairdressers (OR = 1.3, p<0.05, Table 3).

**Table 2:** Prevalence of symptoms, symptom-related adjustments of behaviour and severity associated with inhalation of fragranced products in hairdressers and the general population.

	All hairdressers (n = 5239)	General population (n = 4242)	Adjusted OR^1^	95% CI
	% (n)	% (n)		
Ever experienced symptoms	21.5 (1125)	15.2 (643)	1.0	0.89–1.14
At least one of the symptoms below	21.2 (1112)	14.9 (634)	1.0	0.88–1.14
Upper airways	15.1 (792)	12.7 (537)	**0.8**	**0.72–0.95**
Lower airways	10.0 (524)	6.5 (277)	1.2	0.99–1.43
Headache	16.1 (844)	9.9 (421)	1.0	0.88–1.17
Other CNS symptoms^a^	6.0 (312)	2.9 (121)	**1.5**	**1.15–1.90**
Adjustments of social life^b^	1.5 (78)	1.0 (41)	1.5	0.92–2.41
Adjustments of occupational conditions^c^	2.0 (106)	1.7 (72)	1.1	0.78–1.66
Severity group				
Mildly chemically intolerant^d^	18.4 (965)	12.7 (537)	1.0	0.89–1.12
Severely chemically intolerant^e^	2.8 (147)	2.3 (97)	1.1	0.80–1.51

a Grogginess or exhaustion.

b Social life includes 1) public transportation, 2) social functions in the private sphere or 3) gatherings in the public sphere.

c Occupational conditions include 1) sick leave from work or school/college, 2) permanent leave from employment or school/college or 3) inability to work.

d Reporting at least one symptom related to fragranced products but no impact on behaviour.

e Reporting at least one symptom related to fragranced products and at least one adjustment of social or occupational conditions.

1 Adjusted odds ratio for the difference between all hairdressers and the general population (ref), adjusted for sex and age by logistic regression analyses.

**Table 3:** Prevalence of symptoms, symptom-related adjustments of behaviour and severity associated with inhalation of fragranced products in former and current hairdressers.

	Former hairdressers(n = 2321)	Current hairdressers(n = 2918)	Adjusted OR^1^	95% CI
	% (n)	% (n)		
Ever experienced symptoms	22.7 (526)	20.5 (599)	**1.2**	**1.01–1.31**
At least one of the symptoms below	22.4 (519)	20.3 (593)	1.1	1.00–1.31
Upper airways	15.6 (363)	14.7 (429)	1.1	0.93–1.26
Lower airways	11.4 (265)	8.9 (259)	**1.3**	**1.11–1.59**
Headache	17.8 (413)	14.8 (431)	**1.3**	**1.10–1.48**
Other CNS symptoms^a^	6.4 (148)	5.6 (164)	1.2	0.92–1.46
Adjustments of social life^b^	2.0 (46)	1.1 (32)	**1.8**	**1.12–2.80**
Adjustments of occupational conditions^c^	3.1 (71)	1.1 (33)	**2.8**	**1.84–4.25**
Severity group				
Mildly chemically intolerant^d^	18.5 (430)	18.3 (535)	1.0	0.89–1.18
Severely chemically intolerant^e^	3.8 (89)	2.0 (58)	**2.0**	**1.40–2.75**

a Grogginess or exhaustion.

b Social life includes 1) public transportation, 2) social functions in the private sphere or 3) gatherings in the public sphere.

c Occupational conditions include 1) sick leave from work or school/college, 2) permanent leave from employment or school/college or 3) inability to work.

d Reporting at least one symptom related to fragranced products but no impact on behaviour.

e Reporting at least one symptom related to fragranced products and at least one adjustment of social or occupational conditions.

1 Adjusted odds ratio for the difference between ex-hairdressers and current hairdressers (ref), adjusted for sex and age by logistic regression analyses.

A total of 1.5% of the hairdressers and 1.0% of the general population reported at least one adjustment of social conditions caused by fragrance-related symptoms, while 2.0% of the hairdressers and 1.7% of the general population reported at least one adjustment of occupational conditions caused by fragrance-related symptoms. The differences in adjustments of social or occupational conditions between hairdressers and the general population were not significant (Table 2). However, among hairdressers, both social and occupational adjustments were significantly more frequent among former hairdressers compared with current hairdressers (OR = 1.8, CI = 1.12–2.80 and OR = 2.80, CI = 1.84–4.25, respectively, Table 3).

A total of 18.4% of the hairdressers and 12.7% of the general population were classified as ‘mildly chemically intolerant’, whereas 2.8% of the hairdressers and 2.3% of the general population were classified as ‘severely chemically intolerant’. The differences between groups were not significant when adjusting for sex and age (Table 2). Among hairdressers, a significantly larger proportion of the former hairdressers was classified as ‘severely chemically intolerant’ (3.8%) compared with the current hairdressers (2.0%) (OR = 2.0, CI = 1.40–2.75, Table 2).

Characteristics of ‘severely chemically intolerant’ former hairdressers compared with all other former hairdressers are presented in Table 4. No differences were observed in sex and age between the two groups. Several health-related reasons for leaving the hairdressing profession were reported significantly more frequently among ‘severely chemically intolerant’ former hairdressers compared with the remaining former hairdressers (Table 4). These included: allergy (55.1% vs. 14.2%), musculoskeletal pain (53.9% vs. 37.0%), asthma (34.8% vs. 4.2%) and hand eczema (32.6% vs. 20.2%, Table 4). No differences were observed regarding other diseases or pregnancy as health-related reasons for leaving the profession (Table 4). Notably, the group of remaining former hairdressers reported a different pattern of health reasons for leaving the profession (Table 4).

**Table 4:** Distribution of sex, age, health- and work-related reasons for leaving the hairdressing profession and satisfaction with the hairdressing profession among former hairdressers.

	Severely chemicallyintolerant^1^ (n = 89)	Others^2^ (n = 2232)		Adjusted OR^3^	95% CI
	% (n)	% (n)			
Sex				1.0*	†
Women	96.6 (86)		95.7 (2136)	†	†
Men	3.4 (3)		4.3 (96)	†	†
Age				0.1*	†
<30	13.5 (12)		10.1 (226)	†	†
30–39	42.7 (38)		46.3 (1034)	†	†
40–49	40.4 (36)		42.4 (947)	†	†
50–59	3.4 (3)		0.8 (18)	†	†
≥60	0 (0)		0.3 (7)	†	†
Health-related reasons for leaving the profession^a^					
Hand eczema	32.6 (29)		20.2 (451)	**1.9**	**1.22–3.04**
Asthma	34.8 (31)		4.2 (93)	**12.3**	**7.52–19.93**
Allergy	55.1 (49)		14.2 (317)	**7.4**	**4.80–11.45**
Musculoskeletal pain	53.9 (48)		37.0 (826)	**2.0**	**1.30–3.07**
Other diseases	24.7 (22)		18.1 (405)	1.5	0.90–2.41
Pregnancy (physical problems)	6.7 (6)		5.6 (124)	1.2	0.52–2.86
Pregnancy (chemical influence)	2.2 (2)		3.0 (67)	0.7	0.18–3.72
Work-related reasons for leaving the profession^a^					
Late working hours	42.7 (38)		48.0 (1072)	0.8	0.52–1.24
Working on Saturdays	34.8 (31)		37.8 (843)	0.9	0.57–1.39
Scheduling of the weekly day off	2.2 (2)		4.3 (96)	0.5	0.13–2.12
Too much training in evenings and at weekends	10.1 (9)		8.8 (196)	1.2	0.58–2.37
Too many courses in the evening and at weekends	11.2 (10)		9.4 (210)	1.2	0.62–2.39
Low wages	25.8 (23)		29.2 (652)	0.9	0.53–1.40
Inadequate pay for attending training and courses	28.1 (25)		21.6 (481)	1.4	0.90–2.32
Difficulty working with the employer	12.4 (11)		12.9 (289)	1.0	0.50–1.82
Difficulty working with colleagues	5.6 (5)		2.8 (63)	2.1	0.82–5.35
Difficulty working with customers	13.5 (12)		11.3 (253)	1.2	0.66–2.31
Satisfaction with the profession					
Mainly satisfied with working as a hairdresser	92.1 (82)		83.2 (1856)	**2.4**	**1.08–5.15**
Would like to resume hairdressing if possible	60.7 (54)		36.2 (808)	**2.8**	**1.79–4.29**

a It was possible to state more than one reason for leaving the profession.

1 Reporting at least one symptom related to fragranced products and at least one adjustment of social or occupational conditions.

2 No symptom-related adjustment of social or occupational conditions.

3 Adjusted odds ratio for the difference between severely chemically intolerant former hairdressers and other former hairdressers (ref), adjusted for sex and age by logistic regression analyses.

* p-value, Fisher’s exact test.

† Data not available.

The ‘severely chemically intolerant’ former hairdressers did not differ from the remaining former hairdressers regarding sex, age or any of the work-related reasons for leaving the hairdressing profession, which covered working hours, wages and social work relations (Table 4).

Significantly more ‘severely chemically intolerant’ former hairdressers (92.1%) compared with other former hairdressers (83.2%) reported being mainly satisfied with the hairdressing work (OR = 2.4, CI = 1.08–5.15) and would like to resume hairdressing if possible (60.7% and 36.2%, respectively, OR = 2.8, CI = 1.79–4.29) (Table 4).

## Discussion

To our knowledge, this study is the first to investigate the prevalence and the severity of fragrance-related symptoms in hairdressers. Our results are based on two large samples and suggest that hairdressers are not more chemically intolerant than the general population. To date, the evidence of a possible link between chemical exposure and development of MCS has been derived from occupational clinics and environmental health centres and suggests a lack of association since these MCS patients mainly worked in the service industry (e.g. education and health care) or were homemakers [15]–[18]. Although the hairdressers reported ‘other CNS symptoms’ significantly more often than the general population did, they also reported fewer symptoms from the ‘upper airways’ than the general population did. These contrasting results are likely to be random findings as no differences were observed for other symptoms or the severity.

Nevertheless, a fairly consistent pattern in terms of chemical intolerance was found among hairdressers. Former hairdressers reported ‘experiencing fragrance-related symptoms’, symptoms from ‘lower airways’ and ‘headache’ significantly more often than current hairdressers did. Further, significantly more former hairdressers reported behavioural adjustments, especially occupational adjustments, compared with current hairdressers. This was reflected in a significantly larger proportion of former hairdressers, compared with current hairdressers, being ‘severely chemically intolerant’. These findings could possibly be explained by a healthy-worker effect and demonstrate that the hairdressing profession is considerably exposed to fragranced products, causing chemically intolerant individuals to leave the profession.

Among former hairdressers, several health complaints (e.g. allergy, asthma and hand eczema) were reported more frequently as a reason for leaving the profession by ‘severely chemically intolerant’ former hairdressers than the remaining former hairdressers. These associations have been reported by others. Contact allergy has been reported to be positively associated with symptoms triggered by fragranced products [25], whereas IgE allergy has not been associated with fragrance-related symptoms [6], [26]. Since allergy is a broader term that also includes cell-mediated allergy, our results do not conflict with these previous findings. Asthma and hand eczema have also been found to be positively associated with experiencing fragrance-related symptoms [25], [26]. Musculoskeletal pain was also reported significantly more frequently by ‘severely chemically intolerant’ former hairdressers as a reason for leaving the profession than by the remaining former hairdressers. Although no controlled studies have investigated this association, a literature review by Lacour et al [27] reported musculoskeletal symptoms to be one of the predominant symptoms reported by MCS patients. Despite these positive associations, the group of ‘severely chemically intolerant’ former hairdressers was not a distinctive group in every aspect since it was similar to the group of remaining former hairdressers regarding sex, age and all work-related reasons for leaving the profession.

It has been suggested that individuals with heightened awareness or concern regarding environmental chemicals may be more likely to develop MCS [28]–[30]. However, others have suggested that these factors could equally well affect the recognition and diagnosis of MCS rather than its incidence [31]. This increased level of awareness or concern seems not to apply to the group of ‘severely chemically intolerant’ former hairdressers as they reported being more satisfied with their former profession and a larger proportion of the ‘severely chemically intolerant’ individuals expressed a wish to resume their prior job if possible compared with the remaining former hairdressers.

The present study has some possible limitations. Firstly, the questions for the hairdressers and the general population were slightly different. However, we do not consider this to be of critical importance since the questions on fragrance-related exposure and symptoms were similar and the questions on adjustments of social and occupational behaviour were identical. Secondly, only symptoms from exposure to fragranced products were investigated, which implies that only a subgroup of chemically intolerant patients was studied. However, as fragranced products are reported to be a triggering factor for symptoms by 80–100% of MCS patients [17], [19], [20], [22], we believed that the majority of chemically intolerant individuals are captured by this approach. Thirdly, only a limited number of symptoms were investigated, i.e. symptoms from ‘upper airways’, ‘lower airways’, ‘headache’ and ‘other CNS symptoms’. Nevertheless, this seems to be of minimal importance, as approximately 99% of those who reported ‘experiencing fragrance-related symptoms’ in both the hairdresser group and the general population also reported at least one of the four listed classes of symptoms. Lastly, the findings of this study are limited to the hairdressing profession and further studies on other occupations are needed to clarify this apparent incoherence between chemical exposure and chemical intolerance. Nonetheless, our findings add further support to the view that MCS does not result solely from the toxic effects of chemical exposure.

### Conclusions

The prevalence and the severity of fragrance-related symptoms were similar in hairdressers and the general population. Among hairdressers, however, former hairdressers were more affected by fragranced products than current hairdressers were. Although fragrance-related symptoms did not seem to be more frequent among hairdressers, the hairdressing profession might pose a problem for those who are chemically intolerant.
